# Soil-Specific Calibration and Integration of Low-Cost Capacitive Soil Moisture Sensors into a Solar-Powered Sensor Node

**DOI:** 10.3390/s26133979

**Published:** 2026-06-23

**Authors:** Yakubu S. Zakaria, Sheng Chen, Thomas A. Adongo, Gordana Kranjac-Berisavljevic, Hadi Larijani

**Affiliations:** 1 Department of Agricultural Engineering, School of Engineering, University for Development Studies, Tamale TL1350, Ghana; 2 Department of Engineering, Glasgow Caledonian University, Glasgow G4 0BA, UK; sheng.chen@gcu.ac.uk; 3 Department of Agricultural Mechanization and Irrigation Technology, Faculty of Agriculture, Food and Consumer Sciences, University for Dvelopment Studies, Tamale TL1350, Ghana; aapusiga@uds.edu.gh (T.A.A.); novagordanak@gmail.com (G.K.-B.); 4 West African Center for Water, Irrigation and Sustainable Agriculture, University for Development Studies, Tamale TL1350, Ghana; 5 Department of Computer Science, Glasgow Caledonian University, Glasgow G4 0BA, UK

**Keywords:** capacitive soil moisture sensor, soil-specific calibration, internet of things, solar-powered wireless sensor node, ESP32, constrained linear calibration, MQTT, InfluxDB

## Abstract

Accurate real-time soil moisture monitoring is critical for optimizing water use and ensuring crop health and food security. This study aims to calibrate and integrate low-cost capacitive soil moisture sensors into a solar-powered sensor node for real-time soil moisture monitoring in a loamy sand soil. Three capacitive soil moisture sensors were calibrated in the laboratory under controlled volumetric water content conditions (0–40%) using a constrained linear regression approach. The system was tested in a limited pilot-scale in a drip-irrigated onion field at the IWAD farm, Yagaba (North-East Region, Ghana). The results showed good agreement of the sensor readings with the soil moisture obtained using the gravimetric method (R^2^ of 0.92–0.94, RMSE of 0.40–0.52%, and MAE of 0.35–0.39%) demonstrating the successful transfer of the calibration functions to field conditions. Soil moisture data was successfully monitored and transmitted from the nodes to a LoRa gateway via LoRaWAN (433 MHz) and from the gateway to a Raspberry Pi edge server via Wi-Fi. Data was stored both locally in SQLite on the Raspberry Pi and on the InfluxDB cloud. These results suggest that the developed system, when extensively validated under field conditions, can be used to support decision-making for data-driven IoT-based irrigation scheduling.

## 1. Introduction

Global food demand is projected to increase significantly by about 50–70% by 2050 as the world population approaches 9.7 billion [1,2]. Meeting this demand will require substantial improvements in water use efficiency under irrigated agriculture, which already accounts for approximately 70% of global freshwater withdrawals [3]. In this regard, optimizing irrigation scheduling has become a critical productivity and sustainability strategy [4,5]. An important technology that has emerged and is increasingly explored for optimizing irrigation scheduling is smart irrigation. Smart irrigation is a data-driven irrigation scheduling strategy that relies on data acquired by various sensors deployed to monitor the environment of crops [6,7].

Soil moisture monitoring is an important component of smart irrigation that has been widely applied for optimizing water usage, ensuring crop health and improving crop yields [8,9,10]. According to [10] accurate and efficient monitoring of soil moisture is not only necessary to support sustainable agriculture but to ensure food security. This is because soil moisture is the primary state variable governing plant water availability and is therefore the most direct indicator for triggering irrigation events [10,11]. Continuous, in situ soil moisture monitoring enables irrigation to be triggered precisely when crops experience water deficit, rather than on fixed time-based schedules, potentially reducing water application by 20–40% without yield reduction [6,12]. Dielectric sensing methods, particularly time–domain reflectometry (TDR), frequency–domain reflectometry (FDR), and capacitance-based measurement are the dominant in situ approaches because they provide instantaneous, non-destructive, and continuous measurement [11,13].

Despite their proven high accuracy and reliability, commercial TDR and FDR sensors are prohibitively expensive for many farmers in developing countries [9,14], which affects their ability to make informed irrigation decisions and implement smart irrigation scheduling strategies. In this context, low-cost capacitive soil moisture sensors, which cost less than $3 on the market, have attracted attention as viable alternatives to the high-grade commercial sensors. Over the past few years, several studies have explored the use of these sensors to monitor soil moisture in different soils for irrigation management. For instance, ref. [2] calibrated and integrated the low-cost capacitive sensors (SKU: CE09640) into an Arduino-based system to monitor soil moisture in a dystrophic red latosol in Brazil. The results of field validation carried out in a kale (*Brassica oleracea var. acephala*) field showed a strong inverse correlation (R < −0.964), high fit (R^2^ > 0.95), and acceptable accuracy (RMSE < 0.05) compared to gravimetric method. Similarly, ref. [15] calibrated and integrated the low-cost capacitive soil moisture sensor (DFRobot v1.0) into a solar-powered soil moisture monitoring system to efficiently manage irrigation water under upland crops. The performance of the capacitive soil moisture sensor was compared with that of the MP306 soil moisture sensor and the results showed close agreement.

Ref. [16] designed, calibrated and evaluated the performance of a low-cost soil moisture sensor (SEN0193) in a sugarcane field under a sub-surface drip irrigation system in India. Performance comparison of the calibrated capacitive sensor with the commercial SM150T indicated strong agreement between the readings recorded by the two sensors. Other studies conducted by [17,18,19] demonstrate that the low-cost capacitive sensor can be used to monitor soil moisture and reliably manage irrigation under different crops.

However, according to [8,18] these sensors require rigorous calibration and validation in specific soil textures in order to ensure their reliability. In Sub-Saharan Africa, the calibration and validation of capacitive sensors for specific soil textures have not been well-explored leaving questions regarding how they perform in specific soil types and field conditions in Sub-Saharan Africa unanswered. This is critical because the dielectric response of a capacitive sensor is strongly mediated by soil texture, bulk density, and organic carbon content [18,20], and applying cross-soil calibration functions to different soils can introduce systematic errors exceeding 8–12% volumetric water content [20].

In recent years, a growing number of studies have explored the integration of capacitive soil moisture sensors into soil monitoring systems to support smart irrigation [15,17,18,19,21]. The use of low-power wide-area network (LPWAN) technologies, such as LoRa, offer transmission ranges of 2–15 km in line-of-sight conditions with sub-milliampere average current consumption, enabling battery-powered sensor nodes that can operate for months without charging under solar power [7,22,23]. Table 1 presents a summary of previous studies on soil monitoring systems and a comparison of the technologies employed.

Considering that the cost of sensors is one of the key barriers to the adoption of smart irrigation by many farmers in Sub-Saharan Africa, a soil monitoring system that incorporates the low-cost capacitive soil moisture sensor and LoRaWAN technology can substantially contribute to increasing crop productivity and water use efficiency in irrigated agriculture.

This paper aims to calibrate and integrate capacitive soil moisture sensors into a soil monitoring system to support soil moisture monitoring under irrigated field conditions in Northern Ghana. Specifically, we seek to answer the following questions: (1) How accurately will the low-cost capacitive soil moisture sensor perform when calibrated using the constrained linear regression approach and soil-texture-specific calibration? (2) How does the laboratory-derived calibration function transfer to field conditions where factors, including spatial soil variability, temperature fluctuation, and installation effects, introduce additional uncertainty?

This work seeks to answer the specific questions formulated above. The paper provides valuable insights and evidence to support and guide the calibration of the low-cost capacitive soil moisture sensor for loamy sand soils in areas with similar soil and agroecological conditions.

## 2. Materials and Methods

### 2.1. Study Site and Soil Characterization

**Figure 1:**
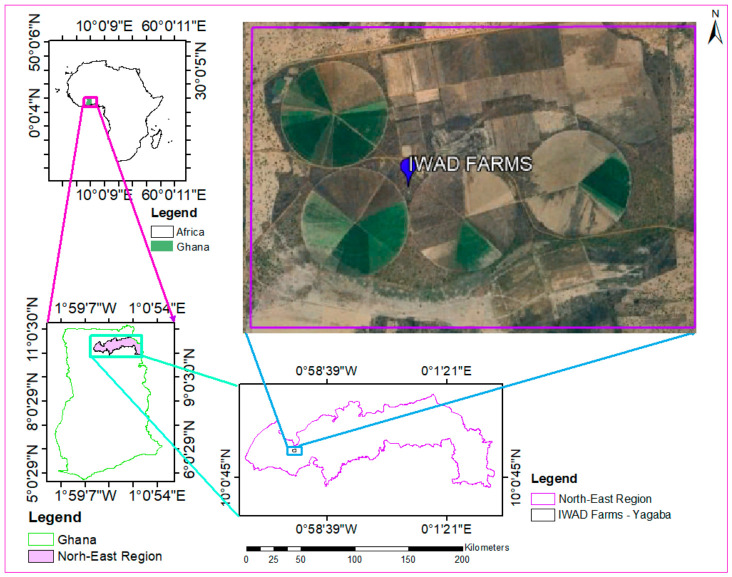
Location of study area.

The study was conducted at the Integrated Water and Agricultural Development (IWAD) farms (10.39° N, 0.50° W; elevation 180 m a.s.l.), located in Yagaba, Mamprugu Moadugri District, North-East Region, Ghana (Figure 1). The site lies within the Guinea Savanna agroecological zone, characterized by a unimodal rainfall regime (April–October, mean annual rainfall 900–1100 mm), a pronounced dry season (November–March), and high potential evapotranspiration (approximately 1600 mm year^−1^). Mean daily temperatures range from 24 °C in January to 38 °C in March. Soils are predominantly loamy sand, developed on Precambrian basement complex parent material.

Surface and sub-surface (0–15 cm and 15–30 cm) soil samples were collected from the experimental field and analyzed at the WACWISA laboratory, University for Development Studies, between June and August 2025, following standard laboratory procedures. Soil properties analyzed include particle size distribution (PSD) using the Bouyoucos hydrometer method and soil dry bulk density (BD) using the core method. Soil pH and electrical conductivity were determined in water (1:2.5) and organic carbon (OC) was determined using the Walkley–Black method. Organic matter (OM) was calculated from OC by multiplying it by a factor of 1.724. Soil moisture at field capacity (FC), permanent wilting point (PWP), saturated water content (SAT), available water content (AWC) and saturated hydraulic conductivity (Ksat) were estimated from PSD and BD using the soil water characteristics software. The key physicochemical and hydraulic properties are summarized in Table 2. The low clay content (2.94–4.12%), high sand fraction (82.5–82.8%), low organic carbon (0.5–0.6%), and high saturated hydraulic conductivity (101–116 mm h^−1^) confirm the coarse-textured, rapidly draining character of the soil, which is consistent with the known dielectric behavior of sandy media and informed the calibration approach adopted in this study.

### 2.2. Sensor Node Design and System Architecture

#### 2.2.1. Hardware Design

**Figure 2:**
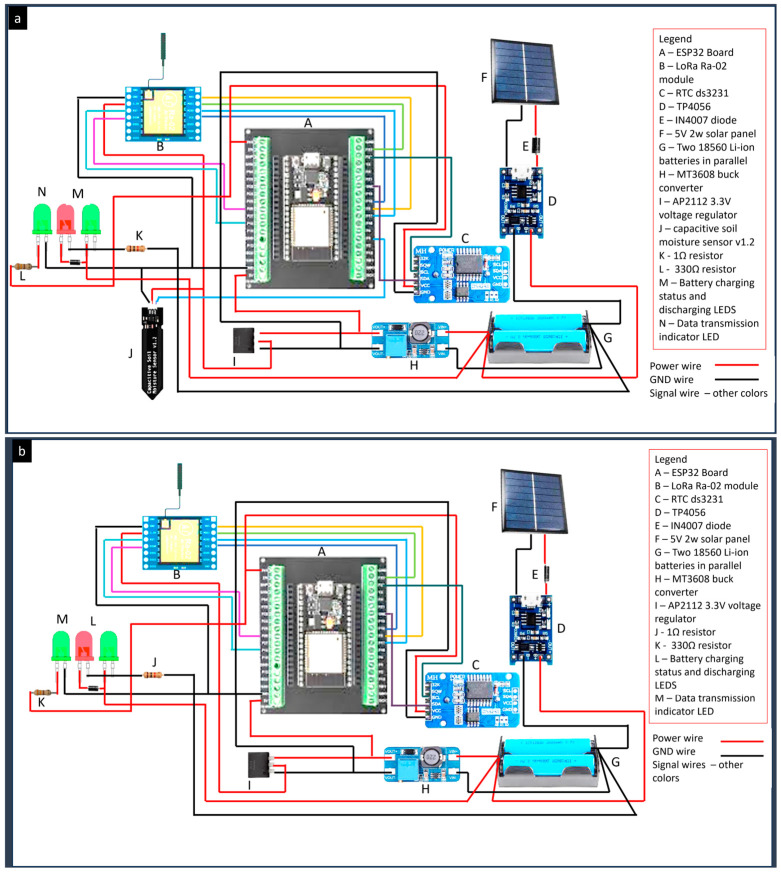
(**a**,**b**) Wiring diagram of the transmitter and receiver.

The soil moisture monitoring system comprised of three sensor nodes and one gateway, which synched soil moisture data via Wi-Fi to a Raspberry Pi edge computing server. The components of the sensor nodes comprised a capacitive soil moisture sensor (v1.2, Analog), an ESP32-WROOM dual-core microcontroller, a LoRa RA-02 transceiver module (433 MHz), a DS3231 real-time clock module, a 6 V/2 W monocrystalline solar panel, two parallel-connected 18,650 Li-ion cells (3.7 V, 2000 mAh each; total capacity 14.8 Wh), a TP4056 Li-ion charge management module with over-discharge protection, and an MT3608 DC-DC boost converter providing regulated 5 V supply. All components were housed in an IP65-rated weatherproof junction box (150 × 110 × 70 mm). The gateway was identical in hardware except for the omission of the soil moisture sensor. The wiring diagram of the sensor node is shown in Figure 2. The costs per unit of the developed sensor node and gateway were approximately USD 63.82 and USD 61.32, respectively. All electronic components were sourced from Oku electronics, Accra, Ghana (https://okuelectronics.com).

**Figure 3:**
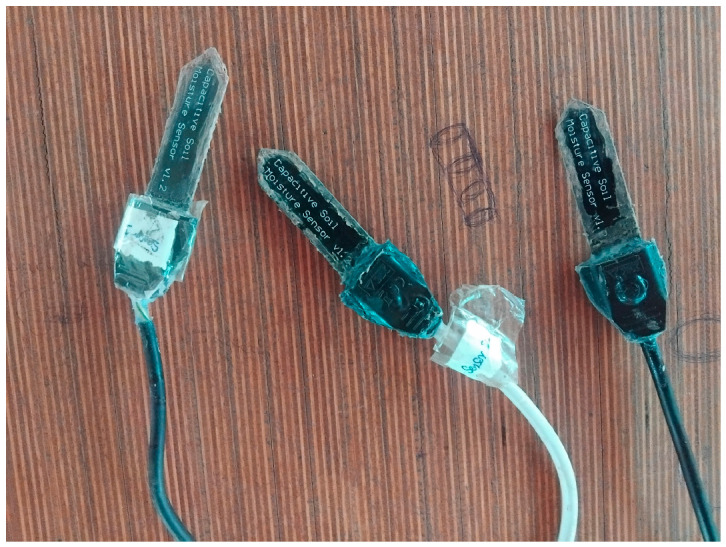
Encapsulation of the capacitive soil moisture sensor in a waterproof protective case.

The choice of the soil moisture sensor was influenced by its low-cost and availability in the local market. LoRaWAN was used due to its low power consumption, wide transmission coverage and to remove the barrier of high data charges associated with Wi-Fi and GSM-based systems. The ESP32 microcontroller was used as the main processing unit due to its low power consumption and integrated wireless communication capabilities, which facilitate its use in IoT-based monitoring systems. A critical design issue associated with capacitive moisture sensor v1.2 is the exposure of its electronic components on the sensor probe body, making it unsuitable for installing it below the soil surface without modification [14]. For this reason, each sensor was encapsulated in a custom protective housing made from a rewireable plastic type C power plug. A clear silicone sealant (GomaGom 58, GomaGom, Bacelona, Spain) was applied on the electronic components and edges of the capacitive soil moisture sensor before encasing it in the protective housing. After firmly enclosing the protective housing with super glue, the silicone sealant was applied on the edges to fully seal any remaining openings creating a waterproof probe that can withstand continuous use under field conditions (Figure 3). Before encapsulation, the signal and the ground pin of the sensor was bridged with a 1 mega ohm resistor to improve the stability of ADC output and avoid drifting of voltage when the sensor is not inserted in the soil. This helped in getting a stable baseline reading and ensured response consistency of the sensor during the laboratory calibration and validation processes. This was necessary because on majority of these sensors, the on-board 1 M ohm resistor connection is often defective leading to unstable ADC readings and voltage drifting.

#### 2.2.2. System Configuration and Node Architecture

**Figure 4:**
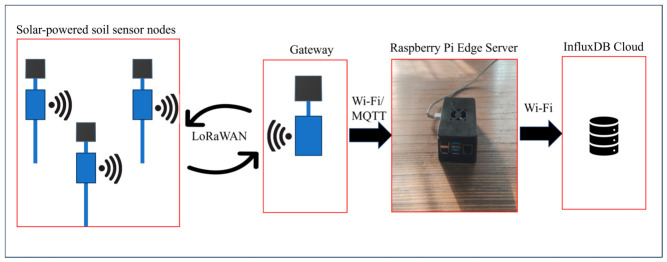
System configuration and communication architecture.

**Figure 5:**
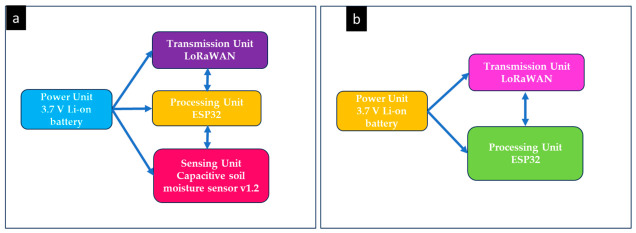
Architecture of the; (**a**) sensor node and (**b**) gateway.

The system is configured to remotely monitor and collect soil moisture data. Three sensor nodes are installed in the field monitor and take soil moisture readings and transmits same via LoRa LoRa (433 MHz, spreading factor SF7, bandwidth 125 kHz, coding rate 4/5) to the gateway. At the gateway, the received signal strength indicator (RSSI) and packet delivery rate is computed, added to the payload and transmitted via the message queuing telemetry transport (MQTT) protocol to a Raspberry Pi edge server from where it is saved locally in SQLite database and sent to InfluxDB via telegraph (Figure 4). The architecture of the sensor node and gateway are shown in Figure 5. The node features a power unit that supplies power to the components, a sensing unit to VWC of the soil, a processing unit to run the logic and a transmission to transmit soil data to the gateway. The gateway is composed of a power unit, a processing unit and a transmission unit.

#### 2.2.3. Energy Analysis and Budget

A sensor network consisting of the transmitters and gateway is powered by two parallel connected 18,650 3.7 V 2000 mAh Li-ion batteries recharged by a 5 V 2w solar panel via the TP4056 module. The system is configured for a 15-min duty cycle to save power and extend the battery autonomy. Daily energy consumption was estimated at approximately 1.33 Wh/day. Under average solar radiation conditions (5 h peak), the 2 W solar panel can provide up to 7.5 Wh/day usable energy, which exceeds the system demand and confirms the suitability of the solar power subsystem for long-term autonomous field deployment. Table 3 presents the estimated energy budget of the system.

#### 2.2.4. Firmware and Data Flow

**Figure 6:**
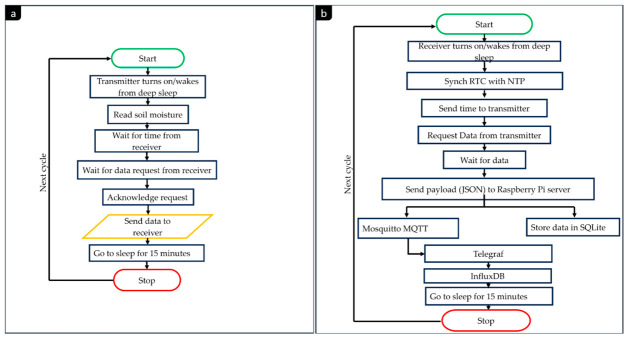
Block diagram of data flow in (**a**) transmitter; (**b**) receiver.

The firmware for the transmitters and the receiver was programmed in the Arduino integrated development environment (IDE) (version 2.3.8) and uploaded onto ESP32 (WROOM) Development Board. The transmission protocol was designed with reliability-oriented features. The receiver polled each transmitter sequentially every 15 min with a 3-retry mechanism on failed acknowledgement. Both the transmitters and the receiver executed a power-optimized deep-sleep cycle between transmissions. Buffering is enabled in the transmitter and receiver to temporarily store data for up to 10 packets when Wi-Fi connection is lost and forward later to the receiver in the case of transmitters and the Raspberry Pi server in the case of the receiver upon reconnection, ensuring that no data is lost during intermittent connectivity. The workflow of the system is shown in Figure 6.

### 2.3. Sensor Calibration: Experimental Design and Statistical Approach

The calibration of all three sensors was performed separately and independently in the WACWISA soil physics laboratory using disturbed loamy sand soil collected from 0–15 cm depth at the drip irrigation blocks at IWAD farms. The study adopted soil-specific, individual sensor calibration to account for the influence of soil texture, bulk density, and organic matter content on the dielectric permittivity–soil–moisture relationships.

Air-dried soil was repacked into seven cylindrical stainless-steel cores (volume 98.19 cm^3^, diameter 50 mm) at a target bulk density of 1.62 g/cm^3^, matching the mean field bulk density. The calibration was done using varying soil conditions, from air-dried soil to saturated conditions. Volumetric water contents (VWC) (θ) corresponding to 5%, 10%, 15%, 20%, 30%, and 40% of the soil volume were prepared gravimetrically by adding corresponding volumes of water to oven-dried soil, thorough mixing, equilibration for 30 min, and repacking. Saturated soil conditions were at 40% VWC. The uncertainty of the VWCs prepared was attributed to the uncertainties associated with the measuring cylinder and the process of repacking the soil into the soil cores. The combined uncertainty was estimated to be ±0.51% VWC for 5% VWC, ±0.53% VWC for 10% VWC, ±0.58% VWC for 15% VWC, ±0.64% VWC for 20% VWC, ±0.78% VWC for 30% VWC, and ±0.94% VWC for 40% VWC. For each of the seven VWCs selected, ten consecutive ADC readings were recorded from each sensor at 2-s intervals via the ESP32 ADC (12-bit resolution, 0–4095). The ten ADC readings were then averaged to obtain a representative ADC value and to mitigate ADC noise for each VWC. The average ADC values for each VWC was used as the calibration dataset.

A constrained linear model was fitted to the sensor ADC response (α, dimensionless) versus θ (%), using the measured dry-soil and saturated-soil ADC values as the minimum and maximum calibration bounds (Equation (1))

(1) VWC=m∗ADC+C

where;

(2) $$m=\frac{\left(\theta_{sat}-\theta_{dry}\right)}{\left({ADC}_{sat}-{ADC}_{dry}\right)}$$

and,

(3) $$c=-m*{ADC}_{sat}$$

$\theta_{sat}$—water content at saturation; $\theta_{dry}$—water content of dry soil; ${ADC}_{sat}$—raw sensor reading of the saturated-soil sample; ${ADC}_{dry}$—raw sensor reading of the dry-soil sample.

The constrained approach has been shown to perform satisfactorily in previous studies [16]. A major motivation for using this approach is to prevent the negative or super-saturated moisture estimates that arise when unconstrained models are extrapolated beyond the calibration range. The performance of the calibration curves was assessed using three metrics: the Nash–Sutcliffe efficiency (NSE), which is equivalent to coefficient of determination (R^2^), mean absolute error (MAE) and root mean squared error (RMSE). The Nash–Sutcliffe efficiency (NSE) quantifies the proportion of observed variance explained by the model:

(4) $$NSE\;=\;1\;-\;[\Sigma {\left(\theta_{\mathrm{si}}\;-\;{\theta^{c}}_{i}\right)}^{2}/\Sigma {\left({\theta^{c}}_{i}\;-\;{\bar{\theta }}^{c}\right)}^{2}]$$

(5) $$RMSE\;=\;\surd [\Sigma {\left({\theta^{c}}_{i}\;-\;\theta_{\mathrm{si}}\right)}^{2}/n]$$

(6) $$MAE\;=\;\Sigma |{\theta^{c}}_{i}\;-\;\theta_{\mathrm{si}}|/n$$

where *θ*_si_ is the sensor-predicted volumetric water content, ${\theta^{c}}_{i}$ is the gravimetrically determined reference value, and ${\bar{\theta }}^{c}$ is the mean reference value over n observations.

### 2.4. Field Testing

**Figure 7:**
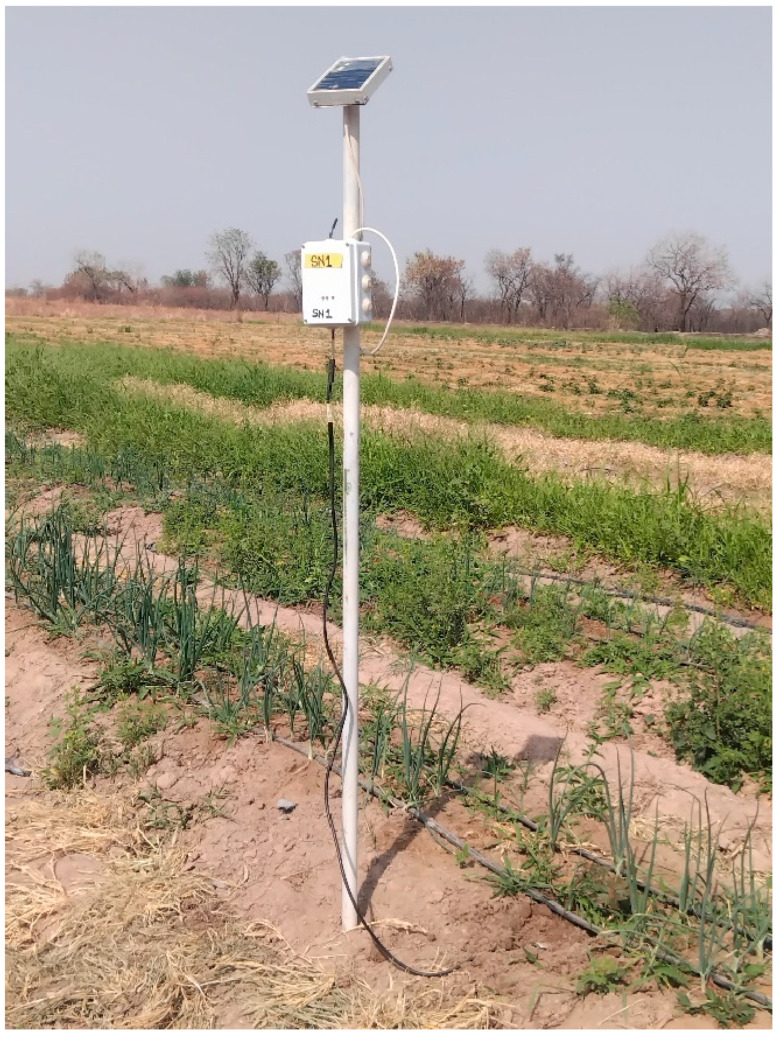
Field testing of the sensor node.

Three sensor nodes and one gateway were tested in a 0.4 ha drip-irrigated onion (*Allium cepa*) field at the IWAD farms in Yagaba (Figure 7) between 27 January 2026 and 11 February 2026. The sensors were installed at 15 cm depth and 5 cm lateral distance from the drip emitter, targeting the primary root zone. Mounting poles positioned transmitters at 1.5 m above ground, with the LoRa antenna extending approximately 8 cm above the top of each housing. During the field testing, the performance of the calibrated sensors was assessed. Also, LoRa transmission range was monitored. Twenty-seven undisturbed core samples consisting of three replicates per sensor location were collected from the field at different locations and the sensor readings recoded at the time of the sample collection. The core samples were tightly covered with aluminum foil and transported to the WACWISA laboratory for gravimetric analysis. After the analysis, the VWC of the samples were then plotted against the sensor readings and the performance assessed based on R^2^, RMSE and MAE.

## 3. Results and Discussion

### 3.1. Laboratory Calibration of Capacitive Soil Moisture Sensors

**Figure 8:**
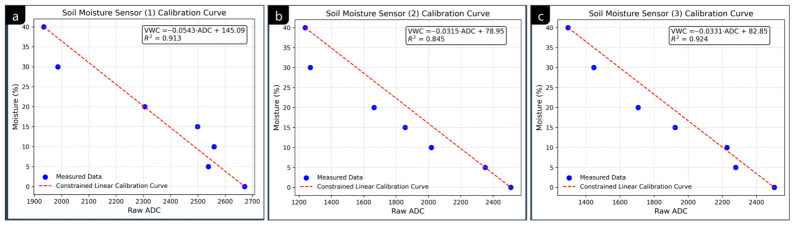
Laboratory calibration curves for the three soil moisture sensors: (**a**) sensor 1; (**b**) sensor 2; (**c**) sensor 3.

The constrained linear calibration curves for all three sensors demonstrated a consistent inverse relationship between the raw ADC output (α) and the gravimetrically determined volumetric water content (θ), as expected from the capacitive measurement principle (Figure 8). As presented in Table 4, the results show that the soil moisture measurements predicted by the calibrated sensors had satisfactory agreement with the baseline soil moisture contents used. The Coefficient of determination (R^2^) ranged between 0.85 and 0.92 while the root mean-square error (RMSE) ranged between 3.60% and 5.14%. The slightly lower R^2^ for sensor 2 (0.85) is attributable to a marginally greater inter-reading variability in its ADC output (standard deviation ±17 counts across the 10-reading windows, versus ±11–13 counts for sensors 1 and 3), likely reflecting minor production variability in the sensor’s oscillator circuit. These results are in line with [8] who reported a satisfactory prediction accuracy with R^2^ between 0.85 and 0.87 and RMSE between 4.5% and 4.9% for capacitive soil moisture sensors calibrated with a polynomial calibration function. The accuracy achieved by the calibrated sensors in this study confirms the assertion that individual calibration of capacitive soil moisture sensors is an effective approach to improving their prediction accuracy in different and specific soil conditions [8].

### 3.2. Field Testing

**Figure 9:**
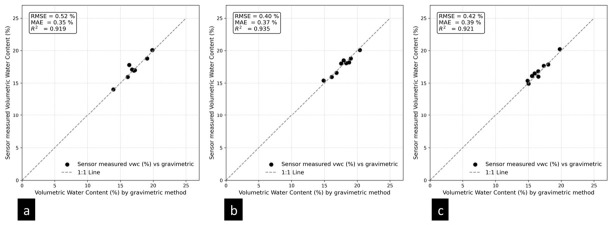
Field validation curves of the capacitive soil moisture sensors: (**a**) sensor 1; (**b**) sensor 2; (**c**) sensor 3.

Figure 9 shows the field performance of the calibrated soil moisture sensors under a limited pilot test. As shown by the figure, the soil moisture measurements recorded by the calibrated sensors achieved satisfactory accuracy with R^2^, RMSE and MAE ranging from 0.92–0.94, 0.40–0.52% and 0.35–0.39%, respectively, when they were compared to the soil moisture values obtained from the 27 gravimetric reference samples taken from the field. These results compare favorably with the accuracy achieved by the sensors under laboratory calibration conditions indicating that field-condition uncertainties were modest and well-controlled by the constrained calibration and installation protocol. The differences in the performance of the sensors during calibration and field test is consistent with laboratory-to-field accuracy disparities reported in previous studies [8,24].

These disparities can be attributed to three identifiable sources of uncertainty. The first one relates to the inherent spatial soil variability at the sub-meter scale, which introduces moisture heterogeneity not captured by a point sensor. The second source is the in situ temperature variation between the time of sensor reading and the time of core sampling. This variation can shift the ADC baseline due to temperature dependence of the oscillator frequency resulting in variations in measured VWC. A one count ADC shift, for instance, can translate to ±0.05% change in VWC (θ) for sensor 1, ±0.031% change in VWC (θ) for sensor 2 and ±0.033% change in VWC (θ) for sensor 3. The third involves minor root disturbance during sensor installation, which can alter the soil bulk density.

The findings highlighted above suggest that laboratory-derived calibration functions can be accurately transferred to the field with minimal uncertainty and without in situ recalibration for loamy sand soils in the study area and other areas with similar agroecological conditions. However, extensive field validation is necessary to further evaluate the performance of the calibrated sensors and ensure their accuracy and reliability for irrigation scheduling applications. For practical irrigation scheduling, the RMSE range of 0.40–0.52% recorded during the field test falls within an operationally acceptable detection uncertainty in triggering irrigation at a given preset decision-relevant threshold or soil-specific trigger point. Based on the measured field capacity water content, permanent wilting point and available water capacity of the loamy sand soil in the study area, a typical irrigation trigger point set at 60–70% of available water capacity depletion corresponds to approximately 6.07–6.36% of volumetric water content. The RMSE range of 0.4–0.52 achieved by the calibrated sensors translates to a relative uncertainty of 6.4–10.5% of the trigger threshold, which is acceptable given that trigger-based irrigation tolerates ±5–10% sensing error without meaningful yield impact [4]. This demonstrates the potential of the sensors to reliably detect irrigation decision points for irrigation scheduling.

**Figure 10:**
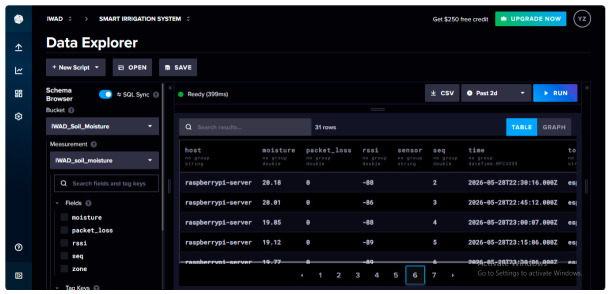
Sample data logged in InfluxDB.

During the field testing, data was successfully transmitted via LoRaWAN from the nodes to the gateway and from the gateway to the Raspberry Pi server and InfluxDB cloud. A screenshot of data explorer dashboard in InflxDB with a sample of logged data is shown in Figure 10.

**Figure 11:**
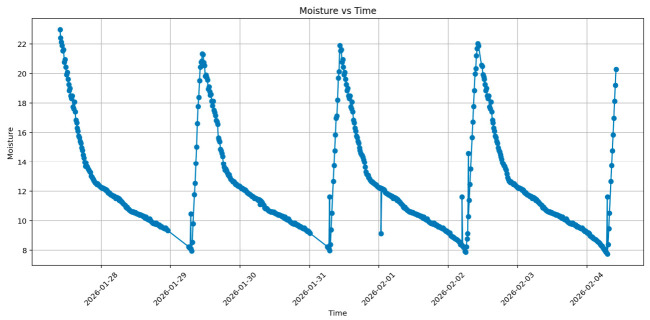
Timeseries plot of data logged by sensor node 1 in the first week of the field test.

The timeseries plot of the soil moisture measurements collected by the sensor nodes from the test field is shown in Figure 11.

#### Challenges Experienced During Field Testing

A major challenge experienced during the field testing of the system was brownouts and power failure caused by supplying power to the LoRa module, the RTC module and the soil moisture sensor from the 3.3 V pin on the ESP32 board. It was observed that on full battery charge, both the node and gateway properly functioned initially for a few hours after which they began to fail as the battery fell below 2.5 V. This was corrected by adding a 3.3 V voltage regulator (AP2112 3.3 V) to supply power to the LoRa module, RTC module and the sensor separately from the ESP32 board. Also, we experienced quicker battery drawdown when the node and gateway was initially configured to run a 5-min duty cycle. Hence, in order to extend the battery life and autonomy, the system was reconfigured for a 15-min duty cycle. This experience highlights the importance of proper power supply configuration and management in such systems to ensure their successful deployment in the field for long term use.

## 4. Conclusions

This study conducted soil-texture-specific constrained linear calibration of the capacitive soil moisture sensor (v1.2) and integrated it into a LoRa-based wireless soil moisture sensor node for remote soil moisture monitoring in a loamy sand soil in Northern Ghana. Field testing of the developed system demonstrated its potential to provide accurate soil moisture measurements to support data-driven irrigation scheduling strategies in the study area. The paper draws the following conclusions:

1. Field testing of the calibrated sensors demonstrated satisfactory performance in the field indicating that the laboratory calibration functions can be reliably transferred to field conditions. This finding suggests that the laboratory calibrated capacitive soil moisture sensors can be directly installed in the field for soil moisture monitoring in loamy sand soils without field calibration, thus making their deployment easy and less time consuming.
2. The developed system was successfully used to remotely and continuously monitor and transmit real-time soil moisture data to InfluxDB cloud suggesting that it can be used to support internet of things (IoT)-based smart irrigation scheduling.

This study provides an evidence-based framework for calibrating and integrating capacitive soil moisture sensors into wireless sensor nodes to support soil moisture monitoring in loamy sand soils in the study area.

### Limitations and Future Research Directions

The calibration functions derived in this study are specific to the loamy sand soils at Yagaba and cannot be applied to soils of substantially different texture or organic carbon content without recalibration.

Future research should consider extending this calibration approach to the sandy loam and clay loam soils found in the Guinea Savanna Agroecological Zone of Ghana. Furthermore, there is the need to conduct extensive studies to validate the performance and accuracy of the constrained linear calibrated capacitive soil moisture sensors and access the robustness, long-term operational challenges, maintenance and replacement costs of the developed soil monitoring system. Additionally, temperature dependance on sensor readings and the long-term drift of the calibration function under different field conditions should be investigated and accounted for in the calibration function. Finally, integration of the developed sensor node and gateway with an automated irrigation controller would enable a fully closed-loop smart irrigation system whose irrigation water savings should be quantified under specific crops in the field.

## Figures and Tables

**Table 1:** A summary of previous studies and the technologies employed.

Study	Location	Communication	Soil Moisture Sensor Type	Power Source	Cal. Method	Board	Data Storage	Parameters
[16]	India	Wi-Fi	Cp v2.1	Battery-SP	G	NODEMCU	AWS	SM
[19]	Tunisia	LoRa sx1276	Cp-EK 1940 v1.2	3.7 V Li-ion battery-SP	G	Arduino Pro mini	Cloud/local	SM
[21]	Nigeria	Bluetooth	FDR	Battery SP	G	Arduino Uno	SD card	pH, SM, T
[17]	USA	LTE	-	Battery	-	Raspberry Pi Zero 2w	-	-
[9]	Vietnam	GPRS-GSM	5TE soil moisture sensor	Battery-SP	-	Arduino Pro Mini	Web-based data server	SM
This study	Ghana	LoRa -sx1278 Wi-Fi	Cp v1.2	3.7 V Li-ion Battey-SP	G	ESP32 Development board	InfluxDB/Local	SM

Cp-capacitive; FDR-frequency–domain reflectometry; SP-solar powered; G-gravimetric; AWS–Amazon Web Services; SM–soil moisture; T-temperature.

**Table 2:** Characteristics of the soils in study area.

**Depth**	**Texture**	**Sand (%)**	**Silt (%)**	**Clay (%)**	**BD (g/cm3)**	**pH**	**EC (mS/m)**	**OC (%)**
**0–15 cm**	Loamy sand	82.8	14.27	2.94	1.62	6.1	4.7	0.6
**15–30 cm**	Loamy sand	82.5	13.38	4.12	1.64	6.0	4.6	0.5
**Depth**	**Texture**	**OM (%)**	**SAT**	**FC (%)**	**PWP (%)**	**AWC (%)**	**Ksat (mm/h)**	
**0–15 cm**	Loamy sand	0.9	42.9	8.1	5.2	2.9	116.0	
**15–30 cm**	Loamy sand	0.9	42.4	8.7	5.4	3.4	101.0	

BD–bulk density; EC–Electrical conductivity; OC–Organic carbon; OM–Organic matter; SAT–Saturation water content; FC–Field capacity; PWP–Permanent wilting point; AWC–Available water content; Ksat–Saturated hydraulic conductivity.

**Table 3:** Energy Budget of the sensor node.

Parameter	Value
Battery capacity	4000 mAh @ 3.7 V
Battery energy	14.8 Wh
Daily power consumption	1.33 Wh/day
Usable solar charge (5 peak hours)	7.5 Wh/day
Daily surplus	6.17 Wh/day
Battery autonomy	11 days

**Table 4:** Laboratory calibration performance metrics for the three capacitive soil moisture sensors.

Sensor	R^2^	RMSE (%)	MAE (%)	Model
Sensor 1	0.91	3.85	2.72	θ=−0.0543∗ADC+145.09
Sensor 2	0.85	5.14	3.80	θ=−0.0315∗ADC+78.95
Sensor 3	0.92	3.60	2.70	θ=−0.0331∗ADC+82.85

θ–volumetric water content.

## Data Availability

The data that support the findings of this study are available from the corresponding author, [Y.S.Z], upon request.
